# Supplementary material for: Role of MicroRNA-502-3p in Human Diseases
Source: Pharmaceuticals (Basel). 2023 Apr 2;16(4):532. doi: 10.3390/ph16040532 (PMC10144852; doi:10.3390/ph16040532)
Supplement: Supplementary file 1 [file pharmaceuticals-16-00532-s001.zip › pharmaceuticals-2294491-Supplementary Table S1.pdf]

MicroRNA-502-3p	Predicted target gene	3P-seq tags	Cumulative score	Total context++ score
hsa-miR-501-3p	TMEM213	5	-0.84	-0.84
hsa-miR-501-3p	RPRD1B	1140	-0.78	-0.78
hsa-miR-501-3p	TMSB15A	14	-0.77	-0.77
hsa-miR-502-3p	KCTD9	575	-0.72	-0.72
hsa-miR-501-3p	TMEM170B	489	-0.72	-0.82
hsa-miR-501-3p	ZNF488	61	-0.65	-0.74
hsa-miR-501-3p	MYNN	289	-0.64	-0.74
hsa-miR-501-3p	SUMO1	180	-0.6	-0.67
hsa-miR-501-3p	CLEC4D	5	-0.57	-0.57
hsa-miR-501-3p	SLC26A6	529	-0.55	-0.55
hsa-miR-501-3p	ADAMTS3	5	-0.55	-0.55
hsa-miR-501-3p	SSX1	5	-0.55	-0.55
hsa-miR-501-3p	SAMD12	57	-0.54	-0.54
hsa-miR-501-3p	SSX4	5	-0.53	-0.53
hsa-miR-501-3p	B4GALT5	1000	-0.53	-0.54
hsa-miR-501-3p	SSX7	5	-0.53	-0.53
hsa-miR-501-3p	SSX4B	5	-0.53	-0.53
hsa-miR-501-3p	ATP1B1	656	-0.53	-0.53
hsa-miR-501-3p	CHMP1B	3248	-0.53	-0.53
hsa-miR-501-3p	SSX3	5	-0.53	-0.53
hsa-miR-501-3p	MDP1	1268	-0.53	-0.53
hsa-miR-501-3p	PLK2	689	-0.52	-0.52
hsa-miR-501-3p	SEC63	3181	-0.52	-0.9
hsa-miR-501-3p	LITAF	11008	-0.52	-0.52
hsa-miR-501-3p	PP13439	289	-0.51	-0.51
hsa-miR-502-3p	RNF144A	124	-0.51	-0.51
hsa-miR-501-3p	ADIPOQ	5	-0.51	-0.51
hsa-miR-501-3p	CCR1	10	-0.51	-0.51
hsa-miR-501-3p	TBCC	587	-0.5	-0.57
hsa-miR-501-3p	CLN5	184	-0.5	-0.5
hsa-miR-501-3p	TM6SF1	211	-0.5	-0.51
hsa-miR-501-3p	CYP27C1	5	-0.49	-0.49
hsa-miR-501-3p	SSX2B	5	-0.49	-0.49
hsa-miR-501-3p	SSX2	5	-0.49	-0.49
hsa-miR-501-3p	LDHAL6A	5	-0.48	-0.48
hsa-miR-501-3p	ASPH	3013	-0.48	-0.48
hsa-miR-501-3p	DCLK3	5	-0.47	-0.47
hsa-miR-501-3p	RASSF5	27	-0.47	-0.49
hsa-miR-501-3p	PMAIP1	12503	-0.47	-0.47
hsa-miR-501-3p	CLIC4	226	-0.47	-0.47
hsa-miR-501-3p	ZIM2	5	-0.47	-0.47
hsa-miR-501-3p	JDP2	110	-0.47	-0.77
hsa-miR-501-3p	MPZL3	59	-0.47	-0.47
hsa-miR-501-3p	COL10A1	5	-0.47	-0.47
hsa-miR-501-3p	PNLIPRP1	5	-0.46	-0.46
hsa-miR-501-3p	SEPSECS	279	-0.46	-0.58
hsa-miR-501-3p	DAPK1	92	-0.46	-0.46

hsa-miR-501-3p	OR10D3	5	-0.46	-0.46
hsa-miR-501-3p	MARCKS	188	-0.46	-0.48
hsa-miR-501-3p	TCEAL4	937	-0.46	-0.46
hsa-miR-501-3p	CDK6	1116	-0.46	-0.52
hsa-miR-501-3p	ITK	5	-0.46	-0.46
hsa-miR-501-3p	NAP1L5	28	-0.45	-0.66
hsa-miR-501-3p	PPP3CC	254	-0.45	-0.46
hsa-miR-501-3p	ATXN1	106	-0.45	-0.54
hsa-miR-501-3p	RBMS1	90	-0.45	-0.45
hsa-miR-501-3p	HDHD2	501	-0.45	-0.49
hsa-miR-501-3p	YIPF7	5	-0.45	-0.45
hsa-miR-501-3p	TUSC1	987	-0.45	-0.45
hsa-miR-501-3p	MRPS27	1137	-0.44	-0.45
hsa-miR-501-3p	C8orf33	359	-0.44	-0.47
hsa-miR-501-3p	DCAF4L2	10	-0.44	-0.44
hsa-miR-502-3p	AL953854.2	5	-0.44	-0.44
hsa-miR-501-3p	PHOX2B	5	-0.44	-0.44
hsa-miR-501-3p	PROM2	18	-0.44	-0.44
hsa-miR-501-3p	RP1-228P16.5	5	-0.43	-0.43
hsa-miR-502-3p	SLC25A13	8	-0.43	-0.43
hsa-miR-501-3p	TSHZ3	81	-0.43	-0.43
hsa-miR-501-3p	UBE2H	855	-0.43	-0.44
hsa-miR-501-3p	OLFM4	5	-0.43	-0.43
hsa-miR-501-3p	LIN7C	1701	-0.43	-0.47
hsa-miR-501-3p	COL12A1	482	-0.42	-0.42
hsa-miR-501-3p	ADAP2	16	-0.42	-0.44
hsa-miR-501-3p	BHMT2	297	-0.42	-0.53
hsa-miR-501-3p	FCGR1A	5	-0.42	-0.42
hsa-miR-501-3p	CCDC3	16	-0.42	-0.42
hsa-miR-501-3p	TIPRL	569	-0.42	-0.52
hsa-miR-501-3p	TARDBP	410	-0.41	-0.43
hsa-miR-501-3p	MYCN	73	-0.41	-0.45
hsa-miR-501-3p	DCUN1D5	1486	-0.41	-0.51
hsa-miR-501-3p	ARPP21	5	-0.41	-0.41
hsa-miR-502-3p	ISCA1	5	-0.4	-0.4
hsa-miR-502-3p	GPR63	5	-0.4	-0.4
hsa-miR-501-3p	AADACL3	5	-0.4	-0.4
hsa-miR-501-3p	COX17	40	-0.39	-0.69
hsa-miR-501-3p	HOXD10	182	-0.39	-0.39
hsa-miR-501-3p	LMX1A	5	-0.39	-0.39
hsa-miR-501-3p	SRSF3	2593	-0.39	-0.48
hsa-miR-501-3p	MRPL35	860	-0.39	-0.4
hsa-miR-501-3p	WDR3	1166	-0.39	-0.59
hsa-miR-501-3p	RAI2	46	-0.39	-0.39
hsa-miR-501-3p	ZEB2	48	-0.39	-0.39
hsa-miR-502-3p	SLA2	5	-0.39	-0.39
hsa-miR-501-3p	SVOPL	5	-0.38	-0.38
hsa-miR-501-3p	DNAJB6	420	-0.38	-0.38

hsa-miR-501-3p	TTLL6	5	-0.38	-0.38
hsa-miR-501-3p	ERMN	5	-0.38	-0.38
hsa-miR-501-3p	CRISP2	5	-0.38	-0.38
hsa-miR-501-3p	APAF1	23	-0.38	-0.46
hsa-miR-501-3p	AURKB	57	-0.38	-0.45
